# Identifying assessment criteria for *in vitro* studies: a method and item bank

**DOI:** 10.1093/toxsci/kfae083

**Published:** 2024-08-30

**Authors:** Paul Whaley, Robyn B Blain, Derek Draper, Andrew A Rooney, Vickie R Walker, Stephen Wattam, Rob Wright, Carlijn R Hooijmans

**Affiliations:** Evidence-Based Toxicology Collaboration at Johns Hopkins Bloomberg School of Public Health, Baltimore, MD 21205, United States; Lancaster Environment Centre, Lancaster University, Lancaster, Lancashire, LA1 4YW, United Kingdom; ICF International, 1902 Reston Metro Plaza, Reston, VA 20190, United States; Department of Anesthesiology, Pain and Palliative Care (Meta Research Team), Radboudumc, 6525 GA Nijmegen, Netherlands; National Institute of Environmental Health Sciences, Research Triangle Park, Durham, NC 27709, United States; National Institute of Environmental Health Sciences, Research Triangle Park, Durham, NC 27709, United States; WAP Consulting, Manchester, United Kingdom; Welch Medical Library, Johns Hopkins University, Baltimore, MD 21218, United States; Evidence-Based Toxicology Collaboration at Johns Hopkins Bloomberg School of Public Health, Baltimore, MD 21205, United States; Department of Anesthesiology, Pain and Palliative Care (Meta Research Team), Radboudumc, 6525 GA Nijmegen, Netherlands

**Keywords:** critical appraisal, risk of bias, in vitro, NAMs, study quality

## Abstract

To support the development of appraisal tools for assessing the quality of *in vitro* studies, we developed a method for literature-based discovery of study assessment criteria, used the method to create an item bank of assessment criteria of potential relevance to *in vitro* studies, and analyzed the item bank to discern and critique current approaches for appraisal of *in vitro* studies. We searched four research indexes and included any document that identified itself as an appraisal tool for in vitro studies, was a systematic review that included a critical appraisal step, or was a reporting checklist for in vitro studies. We abstracted, normalized, and categorized all criteria applied by the included appraisal tools to create an “item bank” database of issues relevant to the assessment of *in vitro* studies. The resulting item bank consists of 676 unique appraisal concepts from 67 appraisal tools. We believe this item bank is the single most comprehensive resource of its type to date, should be of high utility for future tool development exercises, and provides a robust methodology for grounding tool development in the existing literature. Although we set out to develop an item bank specifically targeting *in vitro* studies, we found that many of the assessment concepts we discovered are readily applicable to other study designs. Item banks can be of significant value as a resource; however, there are important challenges in developing, maintaining, and extending them of which researchers should be aware.

*In vitro* study designs are increasingly prevalent in the toxicological literature. This increase in prevalence is driven by efforts to develop experimental models that are better able to detect adverse effects of environmental exposures and minimize the use of animals in research (Hubrecht and Carter 2019). To support appropriate reuse of *in vitro* data in research and decision-making, the results of an *in vitro* study should be assessable for their validity in the context in which they are being used (Hartung et al. 2019). Peer-review of submitted manuscripts is an important control point in ensuring that published *in vitro* papers are useful, transparent, and credible contributions to the literature.

To address an apparent gap in the availability of appraisal tools to facilitate consistent, comprehensive, and exacting peer-review of *in vitro* studies, a new tool “PRIVAT” (Peer-Review of In Vitro studies Appraisal Tool, previously named “IV-CAT”) is being developed (de Vries and Whaley 2018). The methodology for developing PRIVAT includes a systematic search for existing critical appraisal tools and reporting checklists for *in vitro* study designs, and the creation of an “item bank” database of study assessment issues that are relevant to the *in vitro* context. Item banks can have general utility for informing the future development of critical appraisal tools and have previously been published in support of the development of reporting checklists (Page et al. 2019).

In this article, we present the following: our methods for identifying appraisal tools and reporting checklists for *in vitro* studies; our approach to aggregating, categorizing, and normalizing the assessment criteria presented in those tools and checklists; and the final item bank as a comprehensive, structured database of study assessment issues that have been proposed or used in the literature for assessing the quality of *in vitro* studies. We also conduct some descriptive analysis of the included tools to discern patterns and trends in conventional practice for the appraisal of *in vitro* studies. Finally, we make some recommendations about how our item bank can be used to improve the future development of *in vitro* assessment tools.

We had three objectives. Firstly, to identify a comprehensive set of critical appraisal tools applied in the evaluation of *in vitro* study manuscripts. Secondly, to extract all the assessment criteria from the identified tools and checklists and present them in the form of an item bank that can be a resource for the development of future critical appraisal tools and reporting checklists for *in vitro* studies. Thirdly, to present the item bank development methodology, to allow other researchers to replicate our methods in either extending the present item bank or developing item banks to support the creation of critical appraisal tools for other topic areas.

## Materials and methods

### Open science framework project archive

We created an Open Science Framework project archive for storing data related to the development of PRIVAT (https://osf.io/w4fyp/). Data related to the development of the Item Bank is stored in a project subarchive (https://osf.io/7jwvs/). All supplementary materials (SM) are deposited in the subarchive. Links to relevant subarchive sections are provided throughout this manuscript. A complete guide to SM, including direct links to each item, is provided in the Appendix to this manuscript. The subarchive includes the protocol that was developed to guide the conduct of this study (https://osf.io/zwav8/). The protocol was not formally registered and was modified throughout the project. The present manuscript is intended as the authoritative record for the methods that we followed.

### Strategy for identifying appraisal tools and reporting checklists

#### Search strategy

We searched Medline, PubMed, Web of Science, and Embase during September 2018 for *in vitro* critical appraisal tools, using the search strategies presented in SM01 (https://osf.io/tymcd/). We did not conduct a grey literature search. The search strategies were aimed at identifying systematic reviews and research guidance for *in vitro* contexts. We searched for systematic reviews on the assumption that they are likely to critically appraise included studies and will therefore refer to or include critical appraisal tools or reporting checklists. We searched for guidance on how to conduct *in vitro* studies on the assumption that such guidance is likely to present quality criteria for evaluating a study or checklists for reporting a study. Additionally, we hand-searched the citation lists of included documents for references to appraisal tools or reporting checklists.

Search filters from Shojania and Bero (2001) and Wilczynski et al. (2007) were used to locate systematic reviews in PubMed and Embase, respectively. In order to retrieve systematic reviews in Web of Science, the Wilczynski et al. (2007) filter was adapted and used. The search strings for the *in vitro* study concept and research guidance concept were designed by a librarian (RW) in consultation with the research team. The overall search strategy was validated by whether it could locate 100% of a set of documents (*n* = 8) known to be eligible for the review (SM06; https://osf.io/ntz49).

#### Screening

Due to a high volume of search results, collaborators from the Integrative Health Assessment Branch (IHAB), in the Division of Translational Toxicology (DTT) of the National Institute of Environmental Health Sciences (NIEHS) were engaged to support screening. The IHAB collaborators and their contractors, including ICF International, regularly conduct literature screening for systematic reviews and other projects. Title and abstract screening were conducted independently and in duplicate, supported by a machine-learning algorithm in SWIFT Active Screener (Sciome 2018) that was trained to predict document eligibility based on the decisions made by human screeners. Once a sufficient performance threshold had been reached (95% confidence that an automated decision to exclude would be correct) the algorithm was trusted with excluding documents. Documents were then screened at full text in DistillerSR (DistillerSR Inc. 2019), resulting in a set of included documents that were provided for analysis. The results of this initial screening were then rescreened by CRH for eligibility for the specific goals of developing the item bank.

#### Eligibility criteria

Documents were included in this study when they met one of the following criteria: (1) the identified reference presented or utilized a set of formally specified criteria for appraising postconduct the quality of planning, conduct and/or reporting of an in vitro study, via critical appraisal of study reports (referred to henceforth as “assessment tools”); (2) the identified reference presented a formally specified set of characteristics which should be included in a report of an in vitro study (referred to henceforth as “reporting checklists”). Criterion (1) was applied during title, abstract, and full-text screening. Criterion (2) was applied to hand-search results (i.e. following references to named sources for included assessment tools) only.

Papers were excluded when they were: (1) written in another language than English; (2) guidance documents or standards for the conduct of in vitro studies; (3) reviews or surveys of assessment tools and/or reporting standards for in vitro research (reviews of critical appraisal tools and/or reporting standards). Documents identified as (3) were hand-searched for possible relevant documents.

We excluded conduct guidelines because, unlike appraisal tools, guidance documents only provide implicit criteria in the form of recommendations and advice. Although these implicit criteria can in theory be abstracted from a guidance document, the process is much more subjective than for appraisal tools, and therefore challenging to conduct in a way that yields satisfactorily grounded results. We felt that adequately addressing this subjectivity was beyond the resources we had available, and we therefore chose to exclude guidance documents from our literature review. Although we did not search for grey literature, reporting checklists and appraisal tools published outside of peer-reviewed academic journals were eligible for inclusion if they were referenced by an included document and declared themselves to be applicable to *in vitro* study designs.

### Data abstraction, normalization, and coding

#### Data abstraction

Data abstraction was conducted in duplicate by two investigators (CRH and DD). The following information was abstracted for each tool: (i) bibliographic information; (ii) whether the tool or tools in a document were original (i.e. presented for the first time in the document), an adaptation of an existing tool (i.e. based on a tool originally published elsewhere), or were an unmodified existing tool; (iii) whether tools referred to by documents which presented existing or modified tools were themselves eligible for inclusion in the item bank; (iv) the topic area of the tool; (v) the assessment criteria in the tool; and (vi) whether a tool was published as an appraisal tool in its own right or as part of a systematic review of *in vitro* studies. Referenced tools that were eligible for inclusion were added to the included documents.

#### Criteria normalization and coding

Abstracted assessment criteria (the raw criteria as presented in an included tool) were normalized to remove linguistic differences in how appraisal tools express the core assessment issues in a criterion. This was to make it easier to analyze and categorize the issues that the included appraisal tools are assessing. For the normalization process, two investigators (PW, CRH) reworded the abstracted criteria to remove differences in phrasing between functionally equivalent criteria, removed phrasing related to the mode in which the criterion was expressed, and disaggregated composite criteria into discrete individual criteria. The “items” in our item bank are the disaggregated, linguistically minimalist expression of the core appraisal concepts contained in the criteria we abstracted from the included tools and subjected to our normalization process.

**Fig. 1.**
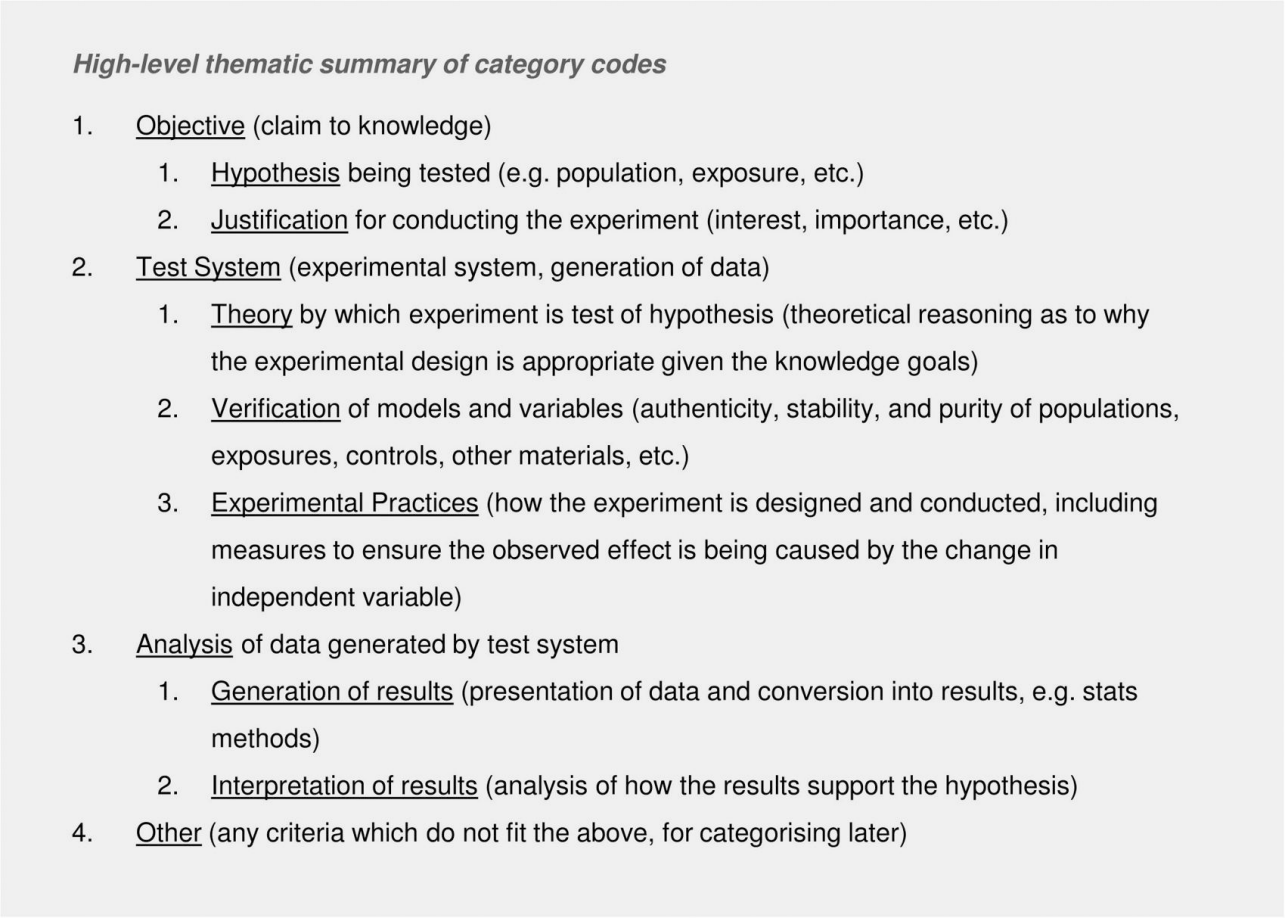
First- and second-level category codes for organizing assessment criteria in the Item Bank.

To organize the items in the item bank, we used a combined deductive and inductive categorization or “coding” strategy. Coding is a method for supporting thematic analysis of qualitative data. In a deductive coding strategy, codes are defined externally to or in advance of the analysis of data. For inductive coding, codes are defined by the investigators while analyzing data, as they observe patterns and themes in data that are useful for the analysis and interpretation of that data. For the PRIVAT item bank, the code structure is hierarchical with up to four levels in the hierarchy. Level 1 codes (e.g. *1 Objective*, *2 Test System*) were defined deductively. Level 4 codes were almost exclusively defined inductively (e.g. *2.3.2.2 Sample size*, *2.3.3.2 Adherence to standardized practices*). Level 2 codes were generally defined more deductively and level 3 generally defined more inductively. The code book is shown in SM02 (https://osf.io/e658q/) and summarized in Fig. 1.

We based our deductive coding strategy on Popper’s hypothetico-deductive (HD) model of how empirical research is conducted (Popper 2002). Popper’s HD model consists of three elements: Conjecture (the hypothesis or prediction being tested); test (the experimental set-up that generates data); and analysis (examination of the data to determine the truth of the conjecture).

For the purpose of coding, we interpreted the main elements of the HD model into conceptual categories relating to the *objectives* of an *in vitro* study (including hypotheses and justification of the research); criteria relating to the design of the *test system* (including e.g. theory by which the experiment tests the conjecture, verification of experimental components, and measures to limit bias); and criteria relating to the *analysis* of data (including how results are generated and then interpreted in terms of their support for the study hypotheses). A fourth level 1 code was added to accommodate *other* criteria (e.g. ethical considerations) that do not fit under the other level 1 codes.

We also coded each assessment item for being presented in “appraisal mode” or “reporting mode” by the tool in which the item appears. “Appraisal mode” means the tool asks its user to make a judgment about the extent to which a quality criterion is fulfilled. “Reporting mode” means the tool only asks if the information needed to make a judgment about the quality criterion has been provided. For example, a criterion of the form “*is the mutation rate in the cell line sufficiently low*” would be coded as being in appraisal mode, whereas a criterion of the form “*did the authors provide information about mutation rate in the cell line*” would be coded as being in reporting mode.

An example of coding and normalization for two criteria is as follows: “*Information regarding the purity of the test chemical should be presented. This may range from a statement that purity was unknown to a detailed analysis. Information on purity strengthens the conclusions drawn.*”; and “*Is the purity of the substance given?*”. These two criteria were both normalized to “Purity of test article” and coded under *2 Test or Experimental System > 2.2 Verification of Experimental Components > 2.2.1 Authenticity of Population*. The former criterion is coded as being in appraisal mode due to connotations about adequacy of purity in relation to drawing conclusions from the study. The latter criterion is coded as being in reporting mode as it only concerns whether information about purity is provided and implies nothing about whether the information is sufficient or the approach taken is adequate.

An example of disaggregating composite criteria is the normalization of “*Statistics: Adequate statistics pre-study and post-study*” to “Pre-study statistics” and “Post-study statistics,” with both coded as being in appraisal mode due to the specific emphasis on judging whether the approach in question is “adequate.”

To maximize interinvestigator consistency in making what were often challenging judgment calls in normalizing tool criteria, coding and normalization were conducted as an iterative process over several rounds. In the first round, criteria were normalized and coded at the first two hierarchy levels by PW and CRH, discussing each criterion in turn. In the second round, PW checked the criteria for consistency, conducted further rephrasing to improve normalization, and defined and applied level 3 and 4 codes. This work was checked by CRH, who highlighted potential errors and issues. In the third round, PW rechecked the criteria for consistency and proposed additional rephrasing in response to the issues identified by CRH. CRH then rechecked the normalization and coding decisions, highlighting any further potential issues with the decisions made. CRH and PW then discussed and resolved issues identified by CRH. Normalization and coding were then halted. The raw abstracted criteria and results of each round of normalization are shown in SM03 (https://osf.io/r3bx6/).

#### Analysis

We conducted descriptive statistical analyses of the data based on frequency counts of individual criteria, and frequencies of criteria within themes.

### Differences between planned (protocol) and applied methods

Rather than use a manual process, we automated removal of duplicate references prior to screening using ICF International’s Python-based Deduper tool (no citation data available). Deduper compares multiple fields (e.g. title, author, year, journal) and can suggest duplicates for manual review even when titles or title-year pairs fail to match. For abstraction and coding, we used Google Sheets instead of DistillerSR. Because of the very large number of study results, the pilot phases for title-abstract and full-text screening were smaller than 5% and 15% of the texts retrieved, respectively. The screening decision-tree and eligibility criteria were modified after pilot-testing to include only SRs and appraisal tools (excluding reporting checklists and guidance documents) when screening the search results. This was to reduce to a manageable level the number of documents that would need to be screened at full-text. The coding strategy and data analysis plans were not described in the protocol.

## Results

We identified 67 appraisal tools applied to *in vitro* studies. After normalization and deduplication, to remove repeat occurrences of a single item from the overall item count, 676 assessment items remained. The items were categorized under 63 assessment codes. The complete list of assessment codes is in SM02 (https://osf.io/e658q). The top-level codes are summarized in Fig. 1. The full-item bank is available as a Microsoft Excel spreadsheet in SM04 (https://osf.io/sar3c). The item bank itself is in sheet 1 of the spreadsheet (“Item Bank”). A visualization of how items are distributed across source appraisal tools is shown in sheet 2 (“Visual Organization of Items”). Sheets 3 to 7 show how we analyzed the data to generate the results discussed below, along with some additional exploratory analyses and heat maps that we have not included in the manuscript. SM03 (https://osf.io/r3bx6) shows the Web of Science topic areas under which the tools have been classified. Sheets 1 and 2 of SM03 list the identifiers and citation information for all of the tools included in the item bank.

### Number of assessment tools

**Fig. 2.**
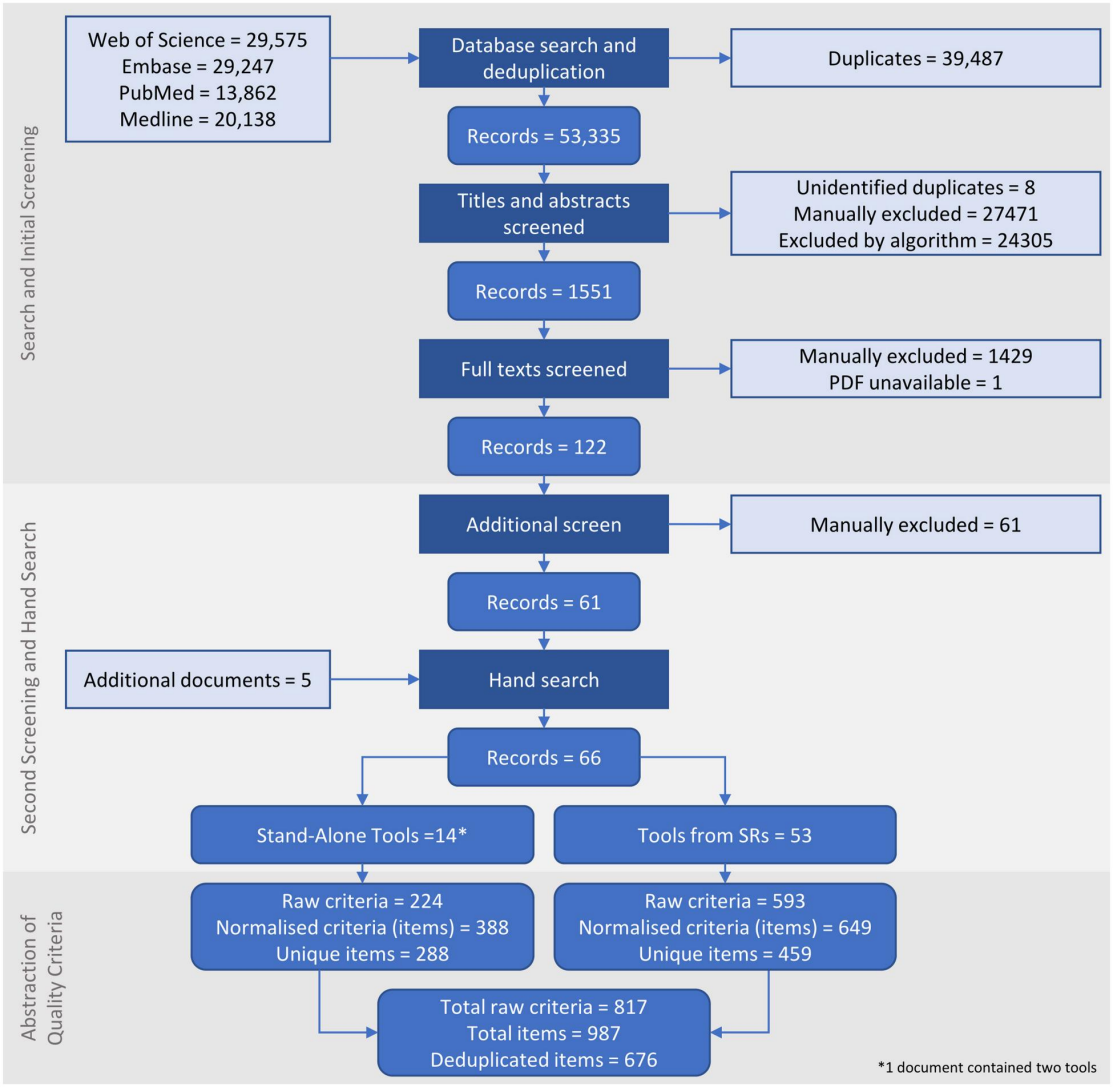
Summary of search for and screening of documents, and abstraction of quality criteria.

The initial screening by the NIEHS contractors (title, abstract, and first full text) yielded 122 documents. The second full-text screening plus hand search by CRH yielded 66 documents of final relevance to the PRIVAT Item Bank (excluding 61 documents but adding 5), one of which contained two tools. In this second round of screening, 25 documents were excluded for either not presenting any assessment criteria, not being applied to *in vitro* experimental studies, or not presenting an original or modified tool. The final set of 67 included tools consisted of 14 that were published as stand-alone tools for assessing in vitro studies (i.e. publications the goal of which was to present an appraisal tool), and 53 tools that were published in SRs that included *in vitro* studies (these are not “stand-alone” tools because the creation of the tool was incidental to the conduct of the SR). The results of the screening process are summarized in Fig. 2.

### Number of assessment criteria and items

**Fig. 3.**
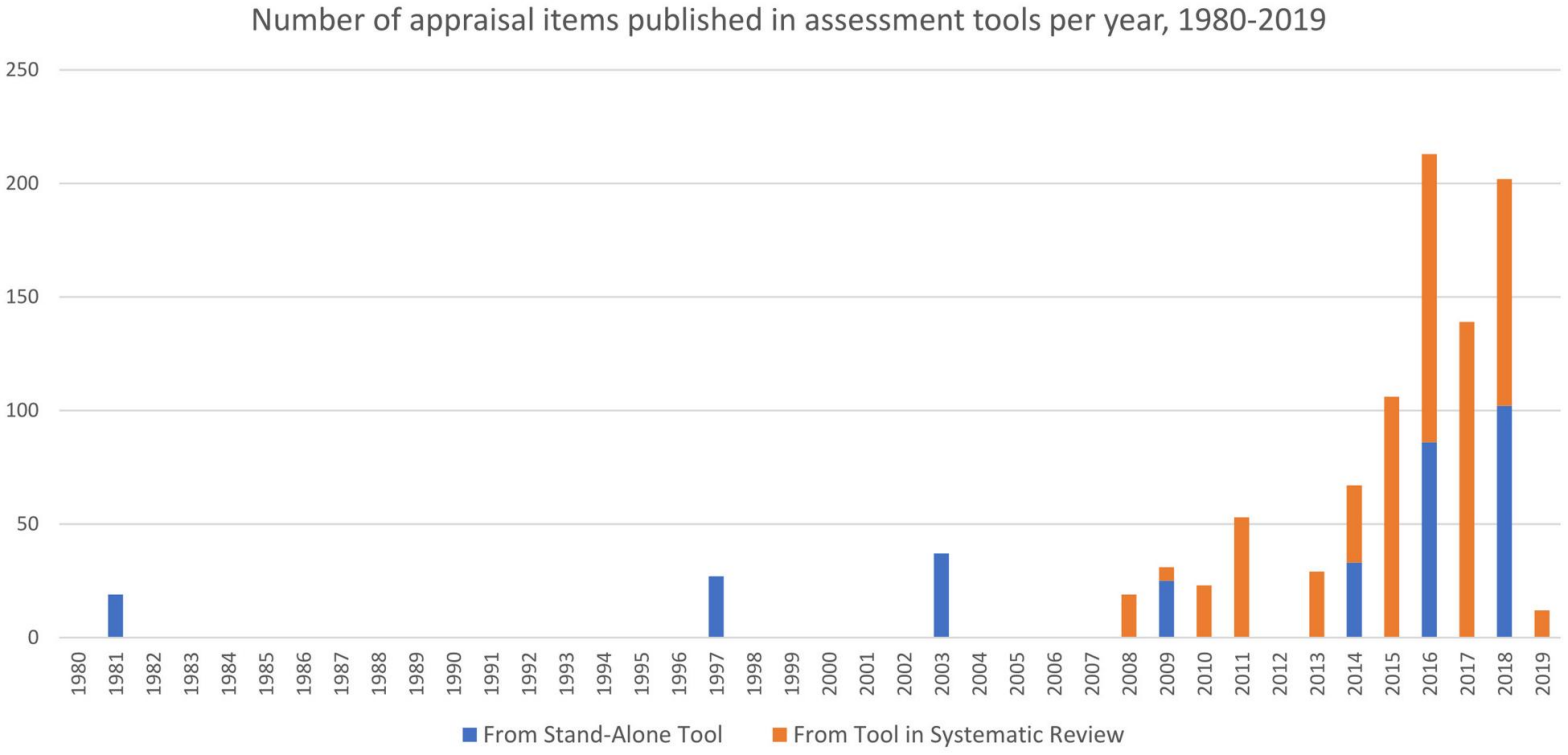
Number of assessment criteria published per year. Our search was conducted in September 2018.

The 14 stand-alone tools presented 224 raw quality criteria. This amounted to 288 items after normalization and deduplication. The tools published as part of SRs presented 593 raw assessment criteria, and 459 items after normalization and deduplication. A combined total of 676 items for the assessment of in vitro studies have been presented in stand-alone appraisal tools and those published in SRs. Most items have been introduced since 2016 (Fig. 3). This coincides with the increase in the publication of systematic reviews in toxicology and environmental health (Menon et al. 2022).

**Fig. 4.**
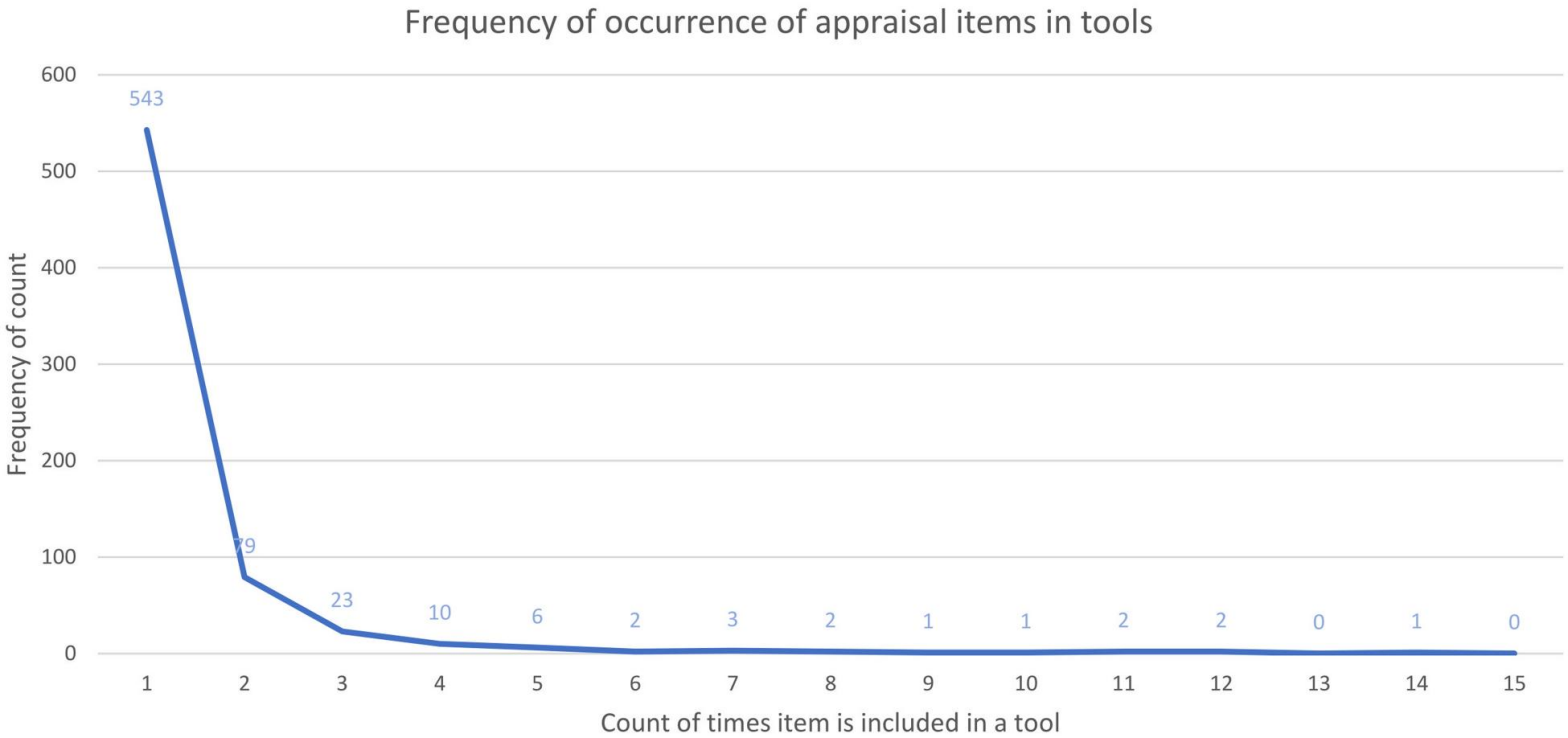
The number of times criteria appear in the included tools. Most criteria only appear once.

Most items (543 of 676, 80%) only appear once in the included appraisal tools (Fig. 4). “Statistical methods” is the most common item, appearing in 15 tools; however, most items appear few times with only 20 of 676 items occurring 5 times or more (SM04, Table S3). Of the 459 items in appraisal tools published in systematic reviews, 393 (86%) only appear once, and only 16 items occur 5 times or more (SM04, Table S5).

### Coverage of assessment concepts

**Fig. 5.**
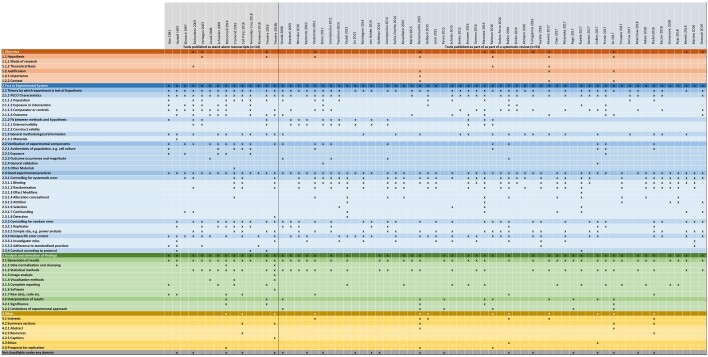
Visual representation of code coverage among included tools. An X indicates a tool has at least one criterion classified as fitting within a code category. An expandable version of this figure is in SM04, Table S2. A detail of the item bank visualization is shown in Fig. 6.

We defined 63 assessment codes to categorize the 676 items. Almost all appraisal tools (96%) addressed issues relating to the experimental system used in a study, and a majority of tools addressed the analysis of data in a study (72%). Fewer tools addressed the objectives of an *in vitro* study (18%). The full set of codes and their position in the code hierarchy are shown in SM02. A full visual array of all categories, criteria under categories, and mode of presentation, as organized by tool, is shown in Table S2 of SM04 (https://osf.io/sar3c). Figure 5 shows a summary of how the assessment criteria for each included tool map onto the code categories.

Twelve of 67 tools in some way assessed (i.e. include at least one item for) the objectives of an *in vitro* study, with a total of 17 items addressing study objectives. Of these, 7 tools in some way assessed the hypothesis of a study (7 items) and 4 assessed the justification for a study (4 items).

Sixty-four of 67 tools in some way assessed the experimental system of a study. Of these, 53 in some way assessed the theory by which the experiment tests the hypothesis (200 items), 20 assessed verification of experimental components (47 items), and 59 assessed how an experimental set-up was protected from error (“good experimental practices,” 185 items). Within good experimental practices, 40 of 67 tools assessed in some way experimental measures for preventing systematic error (66 items, noting that error from e.g. analysis methods are covered under the data analysis categories), and 37 tools assessed in some way controls for preventing random error (23 items).

Forty-eight of 67 tools in some way assessed analysis of data and derivation of findings (118 items). Of these, 47 in some way assessed how results were generated (106 items) and 11 how results were interpreted (11 items). Two code categories that we defined deductively (*1.1.1 Mode of research* and *2.1.2.2 Construct validity*) ended up as empty categories.

**Fig. 6.**
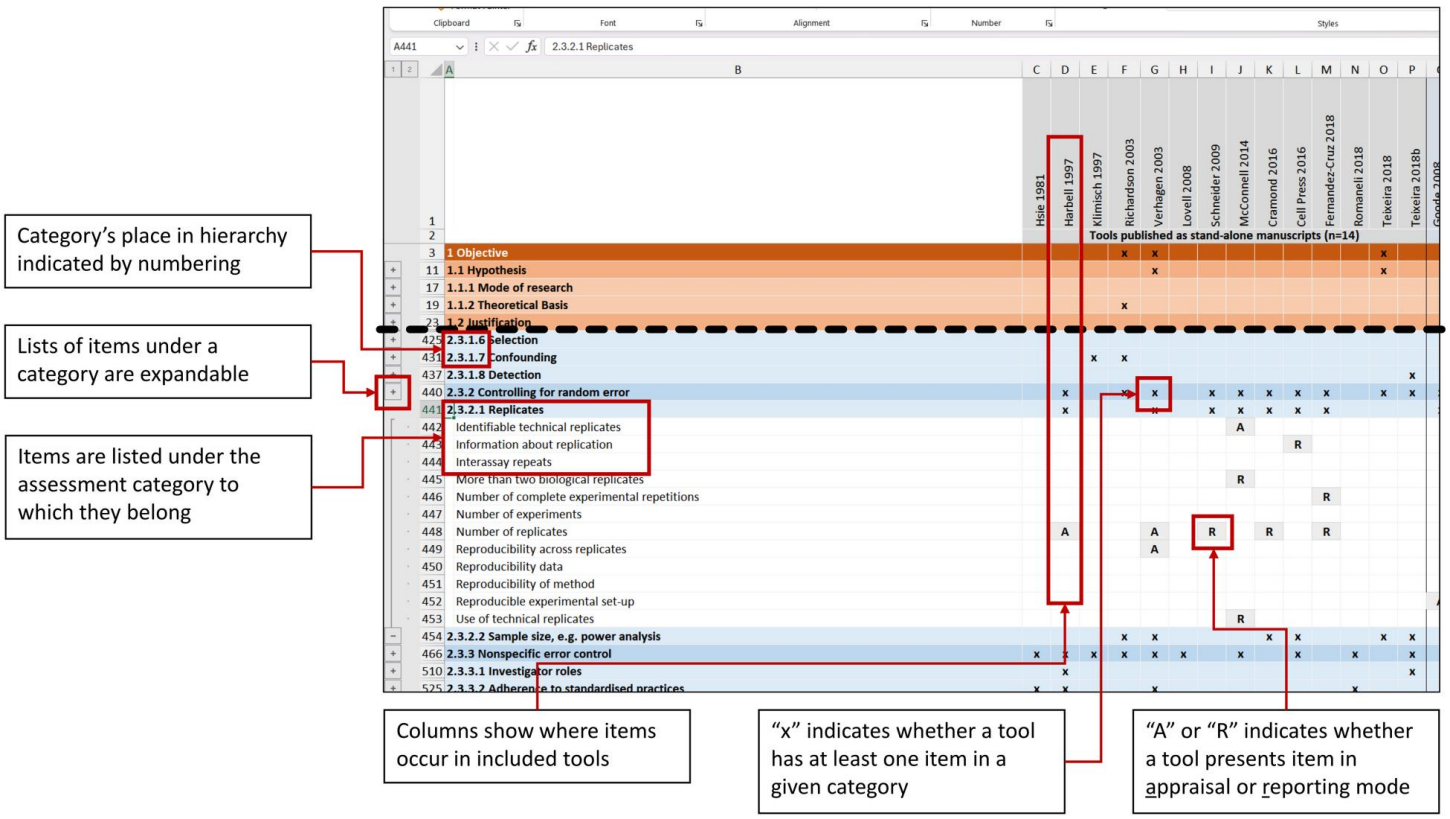
Detail from the visual representation of the item bank. This figure shows how items in the item bank are arranged under a nested hierarchy of categories, how items are related to the tools from which they were abstracted and normalized, and how the distribution of items under categories and across tools is visualized.

Figure 6 shows how the item bank can be used and interpreted. In this figure, the category *2.3.2.1 Replicates*, which is under *2.3.2 Controlling for Random Error*, has been expanded to show that it contains 12 items across 7 stand-alone tools that are in some way covering replicates. The 7 unique items appear a total of 11 times The tools present the items in a mix of appraisal and reporting mode. One item (number of replicates) occurs in five different stand-alone tools. Three tools are only concerned that data about the number of replicates used has been provided. Two tools are concerned with number of replicates being sufficient. One tool (McConnell et al. 2014) includes three items relating to replicates, one of which is presented in appraisal mode and two in reporting, suggesting an inconsistent approach to study assessment in the tool.

**Fig. 7.**
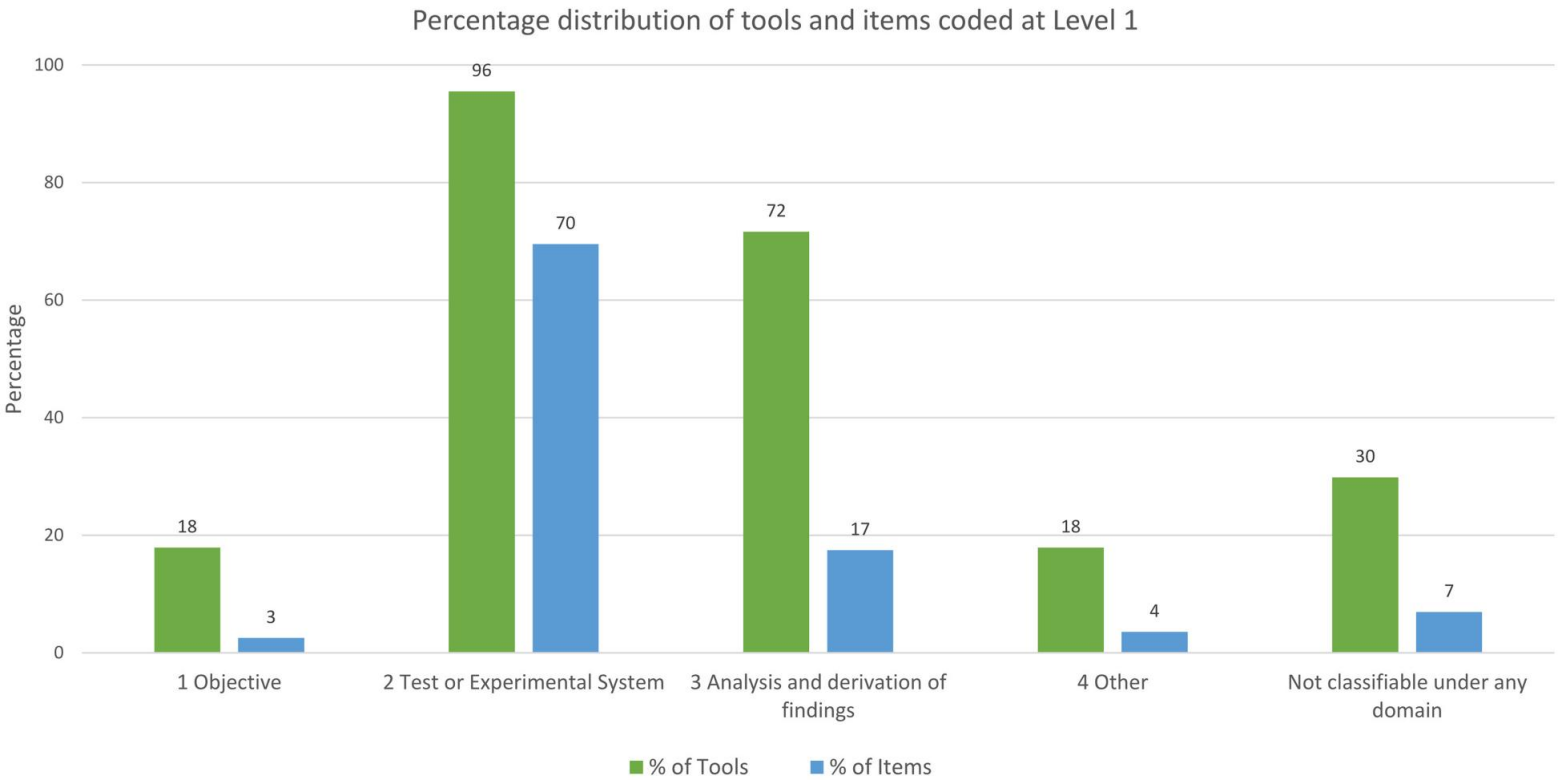
Proportion of tools that include criteria classified under Level 1 codes, and the proportion of items classified under the codes, for stand-alone and SR tools combined. For example, 96% of included tools present at least one item under *2 Test or Experimental System*, and 70% of items are within this category.

Figure 7 shows the distribution of criteria classified under level 1 code categories for all tools. SM04 (Tables S4–S6) drills down into criteria classified under level 2 codes for stand-alone tools, SR tools, and all tools combined.

**Fig. 8.**
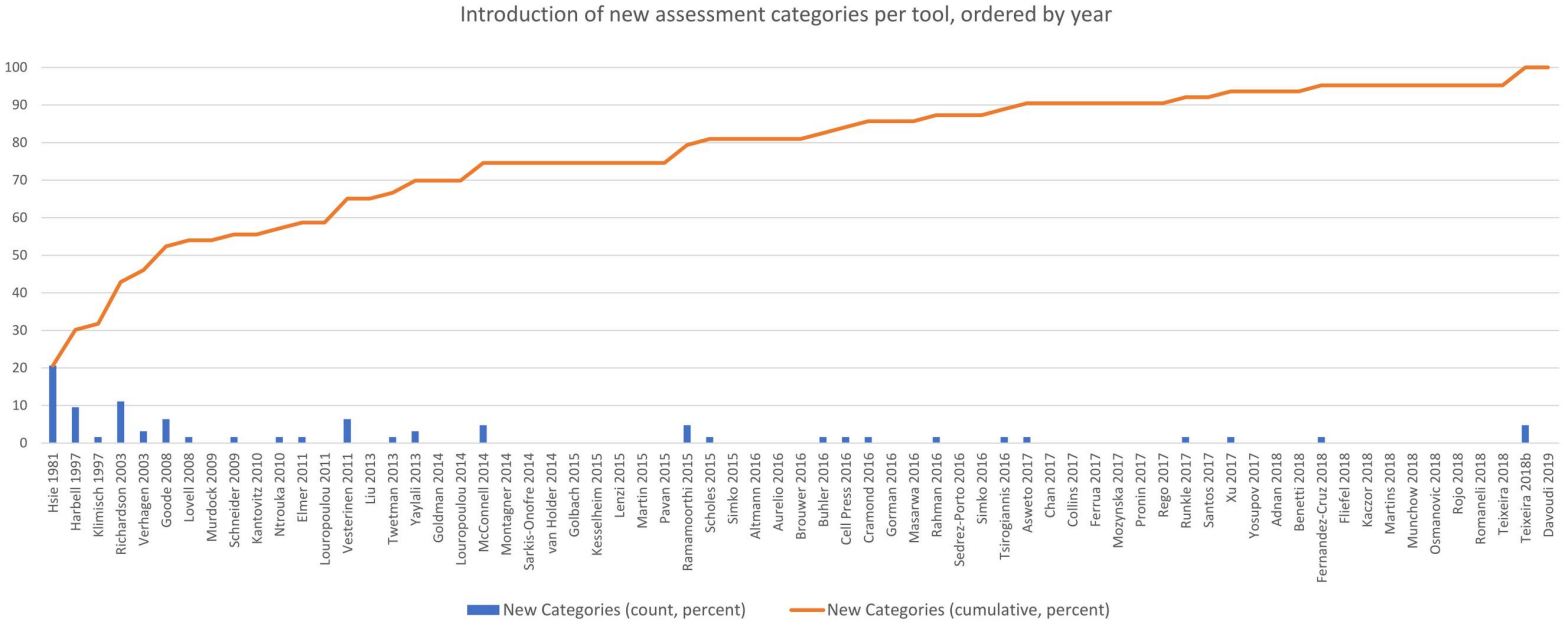
Introduction of assessment categories per tool.

Figure 8 shows the rate of introduction of assessment categories per tool as a percentage over time, with the tools ordered by year of publication and grouped alphabetically within each year. (This is to provide some chronological ordering while accommodating a lack of precise information about when each tool was created.) Seventy-five percent of assessment categories were introduced by the end of 2014. After 2015, only two tools introduced more than one additional assessment category: Ramamoorthi et al. (2015) was the first tool to introduce concepts relating to the justification, importance, and context of a study; and Teixeira and Corrêa (2018) introduced concepts of image analysis, software, and caption accuracy that had not been covered by any previous tools. Two deductively defined codes, *1.1.1 Mode of research* and *2.1.2.2 Construct validity*, had zero criteria and were added from prior knowledge of work by Henderson et al. (2013), Lalu et al. (2019), and Nosek et al. (2018). A list of new assessment categories by tool is in SM05 (https://osf.io/qhu8r).

### Mode of presentation of assessment criteria

**Fig. 9.**
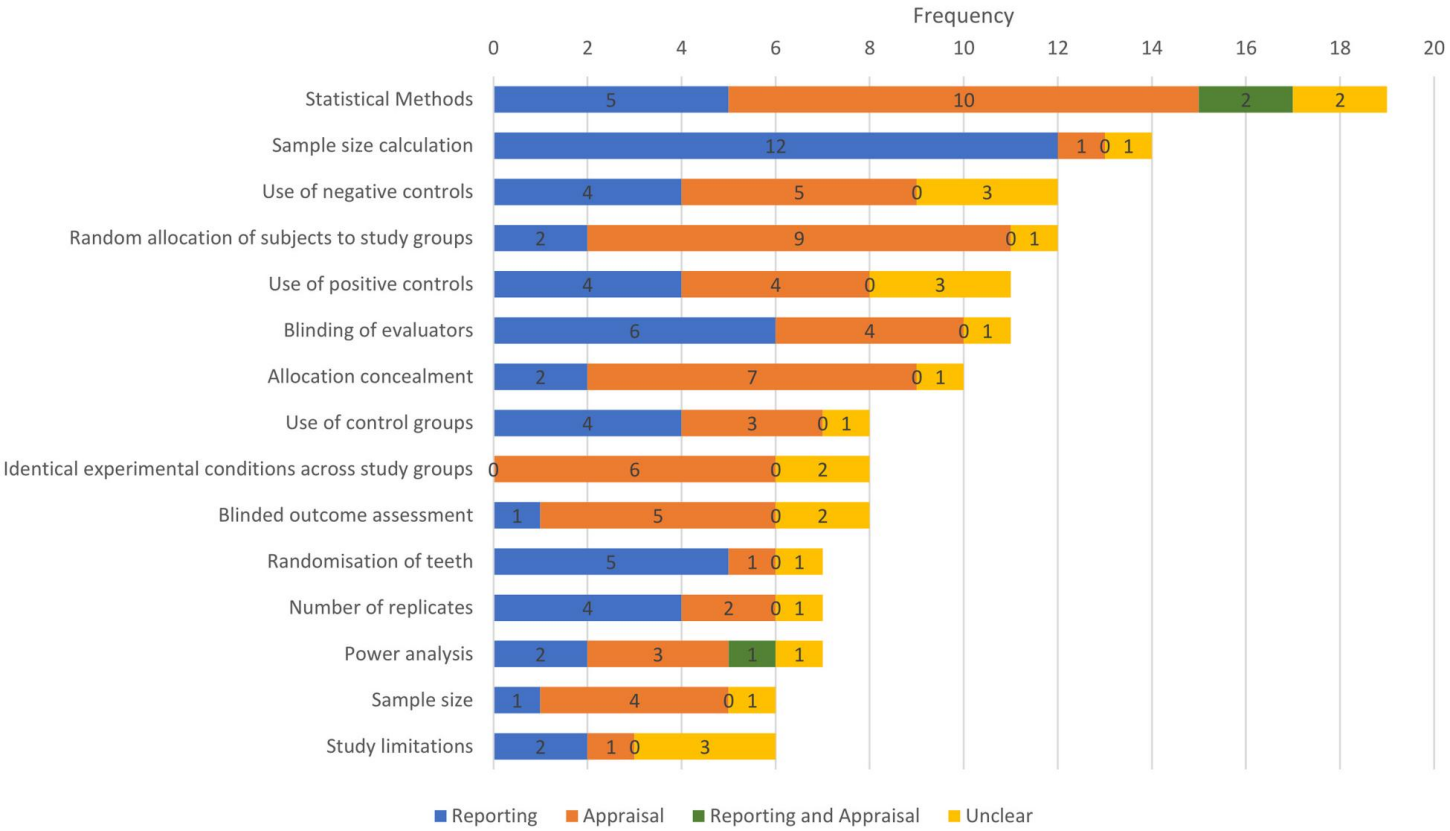
Mode of presentation of the 15 most common assessment criteria.

Figure 9 shows the 15 most commonly occurring items across the included assessment tools and their mode of presentation. Few tools seemed to be unambiguously conceptualized as being an appraisal tool versus being a reporting checklist, with almost all tools including items presented in one or the other of reporting mode and appraisal mode. Some appraisal tools seem to function more as reporting checklists, interrogating whether a given type of information had been provided instead of interrogating how well a study has been conducted in relation to a given quality criterion (see SM03 https://osf.io/r3bx6, Tables S1 and S2). Seventeen percent (170 of 986) of criteria were ambiguously presented, with CRH and PW unable to determine whether they should be coded as being in reporting or appraisal mode.

## Discussion

The aim of this article was to identify a comprehensive set of critical appraisal tools that have been used for evaluating *in vitro* study manuscripts, extract all the assessment criteria from the identified tools and checklists, present the criteria in the form of an item bank, and report the methods for creating the item bank to allow other researchers to apply and extend the approach taken. We identified 67 appraisal tools applied to *in vitro* studies. Fourteen of those tools were published as stand-alone tools for assessing *in vitro* studies, and 53 tools were published within a systematic review. The item bank consists of 676 assessment items under 63 assessment category codes. Most items (80%) occur only once. Although the rate of introduction of new assessment criteria increases rapidly after 2015, the rate of introduction of new assessment categories shelves off at around the same time (Fig. 9).

Our starting assumption in this study was that *in vitro* appraisal criteria would be relatively unique to the *in vitro* domain, which is why (alongside capacity considerations) we set out to develop an item bank specifically targeted at tools within this area of research. However, by conducting this study, we have found that *in vitro* studies can probably be assessed on the same broad principles and structure as other experimental study designs. This is evidenced by how the top-level categories in the organizational hierarchy of our item bank have turned out to be highly generalizable. For example, categories such as *1.1 Hypothesis* and *1.2 Justification* are common to much experimental work.

The differences between *in vitro* and other study designs start to emerge as one moves further down the organizational hierarchy, and arguably it is only in the detail of interpretation of individual criteria that specificity to *in vitro* contexts becomes apparent. Our conceptual framework may therefore provide a general structure for study appraisal that is applicable to many types of study design, whereas the individual items are the part of our work that is more specifically applicable to *in vitro* studies (though some items may have application outside in vitro studies as well).

This lesson has been taken forward by Svendsen et al. in a follow-up study that extends our item bank with additional concepts for assessing the internal validity of *in vitro* studies (Svendsen et al. 2023). The researchers included two human-specific appraisal tools (one for randomized controlled trials and one for epidemiology studies) and found they did indeed have good applicability to *in vitro* contexts, supplying approximately 20% of the total number of internal validity concepts they ended up including in their final dataset (Vist et al. 2024).

### A high degree of variability in individual assessment criteria

At a surface level, the development over the last two decades of assessment tools for *in vitro* study contexts appears somewhat chaotic and unstructured, in terms of both the quality constructs that tools are aiming to appraise and the mode of the appraisal.

The included assessment tools show a high degree of variety in the very specific ways in which they address particular assessment concepts. Eighty percent of items occur only once in the tools included in the item bank, indicating that each included tool tends to have a unique approach to assessing a given appraisal issue.

Items relating to assessment of the blinding of investigators and study participants to various part of the experimental process (category 2.3.1.1) and assessment of sample size (category 2.3.2.2) illustrate how this is happening.

Thirty-six of the 67 tools propose criteria related to blinding, with 18 items across the 36 tools. One tool refers to “blinding” in general, four tools to “blinding of operator of test machines” specifically and exclusively, two to “blinded analysis” specifically and exclusively, and three specifically to “blinded statistical analysis.” 7 of the 18 items concern blinding of particular investigator roles in the study (e.g. operators of machines, personnel in general, evaluators, or caregivers). For sample size (category 2.3.2.2), 29 of the 67 tools propose criteria, with 11 items across the 29 tools.

The need for this level of variety in approach is not obviously explainable. For example, it is not clear to us why blinding of test machine operators should be the only or chief concern when it comes to reducing a study’s risk of bias from observer expectations—as is the case for four tools. Nor is not obvious there are enough differences between contexts to drive as many as 11 different ways of interrogating issues of sample size, nor if all the criteria related to sample size such as “number of specimens” and “sample size calculation” have equal utility or validity when it comes to interrogating whether a study is sufficiently powered.

In terms of mode of appraisal, 23 of the 53 assessment tools from systematic reviews either mixed appraisal with reporting in their assessment, or were unclear in the mode of presentation of their criteria. Fifty-one percent (498 of 986 total items) are presented in reporting mode. Vesterinen et al. (2011) is an unusual example of deliberate interrogation of reporting quality as a proxy for how well a study was conducted.

The level of prevalence of assessment items presented in reporting mode is also difficult to explain. This is because, when assessing the credibility of results of a study in the context of a systematic review, it is generally important to assess how well a given process has been conducted, not just whether the information needed for that assessment has been provided (while the former is mediated by the latter, the two are not equivalent). Yet, of the 508 total items classifiable as being in reporting or appraisal mode for SRs, only 218 (43%) are presented in appraisal mode. Figure 9 and detailed inspection of SM04 (https://osf.io/sar3c, Table S2) show general intra- and intertool inconsistency in the mode of presentation of criteria. Although this is not always the case, the mixture of criteria in reporting and appraisal modes, particularly in assessment tools for systematic reviews, suggests a collective lack of clarity about how study assessment should be conducted.

It is also notable that at least 23 of 53 of the systematic reviews included in our review used or adapted for their critical appraisal process a tool that was not originally designed for *in vitro* studies (SM03 https://osf.io/r3bx6, Table S2). Examples of such tools include: the observational study reporting checklist STROBE (von Elm et al. 2007); the observational study assessment tool MINORS (Slim et al. 2003); the animal study reporting checklist ARRIVE (Kilkenny et al. 2010); and, in the case of GRADE guidance, an assessment process that is not applicable to individual studies at all (Guyatt et al. 2008). Although we believe that appraisal concepts from outside *in vitro* contexts are generalizable to *in vitro* studies, it does not follow that a tool designed for e.g. epidemiology studies can be directly used to assess an *in vitro* study.

### A shared set of thematic issues among assessment tools

Although the examples above indicate that many of the individual decisions about which criteria are appropriate for study appraisal, or which tools should be used or adapted for *in vitro* contexts, may be inappropriate, there is also a substantial degree of concordance among tools: The large majority of items (629 of 676) could be allocated to a thematic domain. This means while each included tool might approach study assessment in a unique specific way, each of the included tools also addresses several of a shared set of general overall themes. This set of themes provides a menu of concepts and criteria that researchers could choose from for their own appraisal tasks, if the researchers can identify a suitable rationale for selection and implementation of these concepts and criteria.

That 629 of 676 items could be allocated to a domain within our conceptual framework also suggests that the majority of study assessment criteria, even those sourced from apparently nonrelevant tools, appear to be plausibly interpretable as applicable to *in vitro* study designs. For example, the items “Randomisation of teeth” and “Representative sample of teeth” might, because they are about teeth, be expected to have poor generalizability to in vitro models, arguably being ex vivo and certainly being specific to dentistry research. Nonetheless, if one disregards the specific reference to teeth, at a general level randomization of sample to experimental group and representativeness of the sample of that which is being sampled have potential applicability to the appraisal of *in* vitro studies. This is not to say that all items are immediately or equally applicable to *in vitro* study designs, just that there may be a greater degree of generalizability of appraisal criteria among specific research topics or disciplines than might otherwise be assumed.

### Using the item bank

Given the evidence that thematic coverage is approaching comprehensiveness, the challenge for appraisal of *in vitro* studies is probably not the discovery of new appraisal themes. Rather, it is to recognize and effectively use the themes that exist while broadening the base of items for use in specific appraisal contexts, especially for themes that are currently underpopulated (e.g. understanding the value or theoretical grounding of a study’s objectives) or could be anticipated to require granular appraisal processes which are new to the *in vitro* domain (such as risk of bias assessment). Given that many tools seem to have been developed somewhat haphazardly, using a methodical approach to identify a comprehensive and thematically coherent set of criteria would appear to be an important guiding principle for any team developing an appraisal tool, to avoid more tools of unclear utility being added to an already large pile.

With regard to tool development, users of our item bank should be aware of how criteria are classified under our HD schema. Our framework divides the research process into the stage of setting an objective, generating data, and analyzing data, ordered around the sequence in which these processes are carried out in a study. It follows that not all items relevant to assessing the internal validity of a study are confined to category *2.3.1 Controlling for systematic error*. This is because code category 2.3 is almost exclusively concerned with experimental practices; for practices that relate to biases introduced by analytical processes (such as incomplete reporting or inappropriate statistical models), the user of the framework will also need to refer to code category *3 Analysis and Derivation of Findings*. Users of the item bank should methodically work through the whole list of concepts and criteria rather than assuming a particular section covers everything that might be needed for their given context.

We would also emphasize that item bank items should not just be used “off the shelf.” For a given assessment task, an assessor will need to select assessment criteria that are appropriate to the assessment goals, then modify these criteria to fit the specific study designs being assessed. Achieving the right balance of granularity and brevity will be challenging. We believe that tool development should involve rigorous expert elicitation and testing processes, and be supported by a strong theory of how the assessment process generates data about study quality that specifically informs the assessment’s goals. A recently developed framework that may help in this regard is the “Focus, Extensiveness, Application, and Transparency” or FEAT framework (Frampton et al. 2022). The FEAT framework emphasizes the need for an assessment tool to focus (F) on quality constructs critical to the assessment goals, to ensure extensive (E) coverage of those constructs, to generate information about those constructs that can be validly applied (A) in data synthesis, and to include sufficient documentation to make transparent (T) the reasons for assessment decisions.

### Limitations, strengths, and extension of the item bank

We have provided a rigorous methodology for deriving from the literature an item bank of study assessment items. In the process, we have generated the most comprehensive list of literature-derived, normalized study quality items created to date. Stopping in 2019, our item bank is not an up-to-date summary of all existing tools. It should also be noted that some conceptual categories still have very few criteria within them. This is particularly the case for the assessment of biological and experimental theory underpinning *in vitro* studies, and for the assessment of external validity of *in vitro* studies. We anticipate these would be the domains under which new assessment categories or subcategories would most likely be introduced.

Because our approach is literature-based, restricted to appraisal tools, and only includes a small number of grey literature sources found by hand-searching of citations, our item bank does not include criteria not mentioned in existing *in vitro* appraisal tools. As a point of comparison, while we identified 66 items under code *2.3.1 Controlling for systematic error*, the draft Scientific Evidence Code System (SEVCO) has identified over 240 bias terms discovered from multiple sources beside study appraisal tools (Alper et al. 2021). Some SEVCO terms relate to bias that can be introduced by early termination of a study, a concept that did not appear in our included tools. Other SEVCO terms offer a much higher level of granularity around how, e.g. selection bias can be introduced to a study besides the five criteria we discovered in our included tools.

Reviews of this kind are time- and labor-intensive, inevitably beginning to miss more recently published studies. That is certainly the case here, with the literature review conducted in 2018. Therefore, on 23 February 2024 PW screened the titles of the first 100 results of two OpenAlex searches (https://openalex.org/), one for “risk of bias tools for in vitro studies” and one for “relevance or reliability of in vitro studies.” This process yielded 2 stand-alone tools: The “QUIN” tool for in vitro studies in dentistry (Sheth et al. 2022) and the SciRAP tool for evaluating the reliability and relevance of *in vitro* toxicity data (Roth et al. 2021). Roth et al. (2021) is being included in an extension of our item bank that targets criteria for the internal validity of *in vitro* studies (Svendsen et al. 2023). Sheth et al. (2022) is a 12 criterion appraisal tool that does not appear to add any thematic issues to our item bank. We are independently aware of the SAToRI-BTR project by Bracher et al. (2020) but as far as we are aware no final tool has yet been published. The OECD’s Guidance Document on Good In Vitro Method Practices (GIVIMP) was published in 2018; however, it provides more of a guide to conducting and reporting in vitro studies than critically appraising them (OECD 2018) and as such would not be eligible for inclusion in our present study. We also found many systematic reviews and other reviews of *in vitro* studies but we did not review them to identify appraisal tools.

In spite of its limitations, to our knowledge our item bank is still the single most comprehensive resource of its type. We included more than twice as many appraisal tools as a recent review by Paiva Barbosa et al. (2023). Another review by Tran et al. (2021) found a similar range of tools as we did (they included the updated SciRAP tool (Roth et al. 2021) and the epidemiology-focused Newcastle Ottawa Scale (Wells 2003), while we included the STAR Cell Press criteria (Marcus et al. 2016) and the assessment tool by Vesterinen et al. (2011)). These reviews neither abstracted any criteria nor provided an organizing framework for appraisal concepts. It is also the first time that a detailed methodology for the creation of an item bank has been published, significantly advancing the approach taken by Page et al. (2019) and greatly reducing the need for tool developers to follow the increasingly unfeasible recommendation of Moher et al. (2014) to systematically review an ever-expanding literature in the course of creating new tools, guidance, and reporting checklists.

In practical terms, our item bank could most readily be extended by adding more reporting checklists (on the assumption that if something should be reported, it represents something that needs to be made known to the reader, and its presence or rigor is therefore a potential target for evaluation) and checklists from guidance documents. Lists from textbooks and grey literature of what researchers should include when articulating the theory behind a study could also be examined. Although we did not attempt to abstract criteria from guidance documents, as we were concerned about our ability to do this comprehensively and objectively, thematic analysis methods supported by experienced qualitative researchers could allow the item bank to be extended with information from these sources.

Researchers intending to extend the item bank should be aware of how challenging it is to build a comprehensive item bank using our methodology. It is not currently possible to define systematic search strategies that retrieve assessment tools with any degree of specificity. We found that to achieve sensitivity, we had to tolerate very low precision of search. (This may change with the maturation of semantic search engines for research and by adding terms for critical appraisal to research indexing systems.)

Once tools have been identified, abstracting and normalizing the criteria they present is time-consuming and, in our opinion, requires a high level of expertise in study appraisal. We have found that the external commitments of researchers with sufficient experience to do the work limit the amount of time that can be spent on the project, resulting in significant delays to completion and explaining why our item bank is not a contemporary summary of the literature. Researchers should be careful to accommodate this when following our methodology.

Given our observation of a significant shelving-off of assessment domains after 2014, it may be valid to use the item bank we present here as a start-point and add concepts and criteria from a purposive sample of additional assessment tools. This approach is being taken by Svendsen et al. (2023) in developing the INVITES-IN appraisal tool for assessing the internal validity of *in vitro* studies. Given the apparently high level of generalizability of assessment tools to contexts outside the topic for which the tool was developed, Svendsen et al. are also including non *in vitro* tools in their sample.

## Conclusion

We developed a methodology for creating a study assessment item bank that yielded 676 assessment items for *in vitro* studies. To the best of our knowledge, this is the single largest resource of its type so far created. Each item in the item bank is classified according to features of methodological rigor under a HD model of research. If applied carefully, we believe the item bank will be useful for informing the development of assessment tools with a range of different appraisal objectives. We also believe our method is replicable, allowing criteria from tools that were not included in our data set to be integrated into the item bank at a future date. Overall, we hope that our work provides a platform of general utility for the future development of *in vitro* assessment tools.

## Supplementary Material

kfae083_Supplementary_Data

## CRediT statement

Paul Whaley (Conceptualisation). Paul Whaley and Stephen Wattam (Formal analysis). Paul Whaley, Carlijn R. Hooijmans, Derek Draper, and Robyn B. Blain (Investigation). Paul Whaley, Carlijn R. Hooijmans, Rob Wright, and Stephen Wattam (Methodology). Paul Whaley, Carlijn R. Hooijmans, and Stephen Wattam (Visualization). Paul Whaley and Carlijn R. Hooijmans (Writing—Original draft). Paul Whaley, Carlijn R. Hooijmans, Rob Wright, Andrew A. Rooney, Vickie R. Walker, and Stephen Wattam (Writing—Review and editing)
