# Supplementary material for: Identifying assessment criteria for in vitro studies: a method and item bank
Source: Toxicol Sci. 2024 Aug 30;201(2):240–53. doi: 10.1093/toxsci/kfae083 (PMC11424884; doi:10.1093/toxsci/kfae083)
Supplement: kfae083_Supplementary_Data [file kfae083_supplementary_data.docx]

**Identifying assessment criteria for *in vitro* studies: a method and item bank**

# ***Supplemental References***

Adnan S, Lone MM, Khan FR, Hussain SM, Nagi SE. 2018. Which is the most recommended medium for the storage and transport of avulsed teeth? A systematic review. Dent Traumatol. 34:59.

Altmann AS, Collares FM, Leitune VC, Samuel SM. 2016. The effect of antimicrobial agents on bond strength of orthodontic adhesives: a meta-analysis of in vitro studies. Orthod Craniofac Res. 19:1–9.

Asweto CO, Wu J, Alzain MA, Hu H, Andrea S, Feng L, Yang X, Duan J, Sun Z. 2017. Cellular pathways involved in silica nanoparticles induced apoptosis: A systematic review of in vitro studies. Environ Toxicol Pharmacol. 56:191–197.

Aurélio IL, Fraga S, Rippe MP, Valandro LF. 2016. Are posts necessary for the restoration of root filled teeth with limited tissue loss? A structured review of laboratory and clinical studies. Int Endod J. 49(9):827–835.

Benetti F, Lemos CAA, e Oliveira Gallinari M, Terayama AM, Briso ALF, e Castilho Jacinto R, Sivieri-Araujo G, Cintra LTA. 2018. Influence of different types of light on the response of the pulp tissue in dental bleaching: a systematic review. Clin Oral Investig. 22:1825–1837. https://link.springer.com/article/10.1007%2Fs00784-017-2278-9.

Brouwer F, Askar H, Paris S, Schwendicke F. 2016. Detecting Secondary Caries Lesions: A Systematic Review and Meta-analysis. J Dent Res. 95:143–151.

Buhler J, Amato M, Weiger R, Walter C. 2016. A systematic review on the effects of air polishing devices on oral tissues. Int J Dent Hyg. 14:15.

Marcus E, whole Cell team. 2016. A STAR Is Born. Cell. 166(5):1059–1060.

Chan H, Ho J, Liu X, Zhang L, Wong SH, Chan MT, Wu WK. 2017. Potential and use of bacterial small RNAs to combat drug resistance: a systematic review. Infect Drug Resist. 10:521–532.

Collins A, Ross J, Lang SH. 2017. A systematic review of the asymmetric inheritance of cellular organelles in eukaryotes: A critique of basic science validity and imprecision. Plos One. 12.

Cramond F, Irvine C, Liao J, Howells D, Sena E, Currie G, Macleod M. 2016. Protocol for a retrospective, controlled cohort study of the impact of a change in Nature journals’ editorial policy for life sciences research on the completeness of reporting study design and execution. Scientometrics. 108:315–328.

A. Davoudi, M. Rismanchian. 2018. Effects of modifying implant screw access channels on the amount of extruded excess cement and retention of cement-retained implant-supported dental prostheses: A systematic review. J Prosthet Dent.

Elmer J, Wittels KA. 2012. Emergency reversal of pentasaccharide anticoagulants: a systematic review of the literature. Transfusion Medicine. 22:108–115.

Fernandez-Cruz ML, Hernandez-Moreno D, Catalan J, Cross RK, Stockmann-Juvala H, Cabellos J, Lopes VR, Matzke M, Ferraz N, Izquierdo JJ, et al. 2018. Quality evaluation of human and environmental toxicity studies performed with nanomaterials - the GUIDEnano approach. Environmental Science-Nano. 5:381–397.

Ferrua CP, Centeno EGZ, Rosa LCD, Amaral CCD, Severo RF, Sarkis-Onofre R, Nascimento GG, Cordenonzi G, Bast RK, Demarco FF, et al. 2017. How has dental pulp stem cells isolation been conducted? A scoping review. Braz Oral Res. 31.

Fliefel R, Ehrenfeld M, Otto S. 2018. Induced pluripotent stem cells (iPSCs) as a new source of bone in reconstructive surgery: A systematic review and meta-analysis of preclinical studies. J Tissue Eng Regen Med. 12:1780.

Golbach LA, Portelli LA, Savelkoul HFJ, Terwel SR, Kuster N, de Vries RBM, Verburg-van Kemenade BML. 2016. Calcium homeostasis and low-frequency magnetic and electric field exposure: A systematic review and meta-analysis of in vitro studies. Environ Int. 92-93:695–706.

Goldman M, Juodzbalys G, Vilkinis V. 2014. Titanium surfaces with nanostructures influence on osteoblasts proliferation: a systematic review. J Oral Maxillofac Res. 5.

Goode A, Hegedus EJ, Sizer P, Brismee JM, Linberg A, Cook CE. 2008. Three-dimensional movements of the sacroiliac joint: a systematic review of the literature and assessment of clinical utility. J Man Manip Ther. 16:25.

Gorman CM, Ray NJ, Burke FM. 2016. The effect of endodontic access on all-ceramic crowns: A systematic review of in vitro studies. J Dent. 53:22–29.

Harbell JW, Southee JA, Curren RD. 1997. The path to regulatory acceptance of in vitro methods is paved with the strictest scientific standards. Animal Alternatives, Welfare, and Ethics. 27:1177–1181.

Hsie AW, Casciano DA, Couch DB. 1981. The use of Chinese hamster ovary cells to quantify specific locus mutation and to determine mutagenicity of chemicals. A report of the GENE-TOX program. Mutation Research. 86:193.

Kaczor K, Gerula-Szymanska A, Smektala T, Safranow K, Lewusz K, Nowicka A. 2018. Effects of different etching modes on the nanoleakage of universal adhesives: A systematic review and meta-analysis. J Esthet Restor Dent.

Kantovitz KR, Pascon FM, Nobre-dos-Santos M, Puppin-Rontani RM. 2010. Review of the effects of infiltrants and sealers on non-cavitated enamel lesions. Oral Health Prev Dent. 8:295–305.

Kesselheim AS, Polinski JM, Fulchino LA, Isaman DL, Gagne JJ. 2015. Modified Regulatory Pathways to Approve Generic Drugs in the US and a Systematic Review of Their Outcomes. Drugs. 75:633–650.

Klimisch H-J, Andreae M, Tillmann U. 1997. A systematic approach for evaluating the quality of experimental toxicological and ecotoxicological data. Regul Toxicol Pharmacol. 25(1):1–5.

Lenzi TL, Gimenez T, Tedesco TK, Mendes FM, Rocha Rde O, Raggio DP. 2016. Adhesive systems for restoring primary teeth: a systematic review and meta-analysis of in vitro studies. Int J Paediatr Dent. 26:364–375.

Liu K, Li Y, Zhang G, Liu J, Cao J, Ao L, Zhang S. 2014. Association between mobile phone use and semen quality: a systemic review and meta-analysis. Andrology. 2:491–501.

Louropoulou A, Slot DE, Van der Weijden FA. 2012. Titanium surface alterations following the use of different mechanical instruments: a systematic review. Clin Oral Implants Res. 23(6):643–658.

Louropoulou A, Slot DE, Van der Weijden F. 2015. Influence of mechanical instruments on the biocompatibility of titanium dental implants surfaces: a systematic review. Clin Oral Implants Res. 26:841–850.

Lovell DP, Omori T. 2008. Statistical issues in the use of the comet assay. Mutagenesis. 23:171.

Martin F, Ufodiama C, Watt I, Bland M, Brackenbury WJ. 2015. Therapeutic Value of Voltage-Gated Sodium Channel Inhibitors in Breast, Colorectal, and Prostate Cancer: A Systematic Review. Front Pharmacol. 6:273.

F. V. Martins, W. F. Vasques, E. M. Fonseca. 2018. How the Variations of the Thickness in Ceramic Restorations of Lithium Disilicate and the Use of Different Photopolymerizers Influence the Degree of Conversion of the Resin Cements: A Systematic Review and Meta-Analysis. J Prosthodont.

Masarwa N, Mohamed A, Abou-Rabii I, Abu Zaghlan R, Steier L. 2016. Longevity of Self-etch Dentin Bonding Adhesives Compared to Etch-and-rinse Dentin Bonding Adhesives: A Systematic Review. J Evid Based Dent Pract. 16:96–106.

McConnell ER, Bell SM, Cote I, Wang RL, Perkins EJ, Garcia-Reyero N, Gong P, Burgoon LD, Guo NL. 2014. Systematic omics analysis review (SOAR) TOOL to support risk assessment. PLoS ONE. 9.

Montagner AF, Sarkis-Onofre R, Pereira-Cenci T, Cenci MS. 2014. MMP Inhibitors on Dentin Stability: A Systematic Review and Meta-analysis. J Dent Res. 93:733–743.

Mozynska J, Metlerski M, Lipski M, Nowicka A. 2017. Tooth Discoloration Induced by Different Calcium Silicate-based Cements: A Systematic Review of In Vitro Studies. J Endod. 43:1593–1601.

Munchow EA, Meereis CTW, e Oliveira da Rosa WL, Silva AF, Piva E. 2018. Polymerization shrinkage stress of resin-based dental materials: A systematic review and meta-analyses of technique protocol and photo-activation strategies. J Mech Behav Biomed Mater. 82:77–86.

Murdock J, Watson D, Doree CJ, Blest A, Roberts MM, Brunskill SJ. 2009. Drugs and blood transfusions: dogma- or evidence-based practice? Transfus Med. 19:6–15.

Ntrouka VI, Slot DE, Louropoulou A, Van der Weijden F. 2011. The effect of chemotherapeutic agents on contaminated titanium surfaces: a systematic review. Clin Oral Implants Res. 22:681–690.

Osmanovic A, Halilovic S, Kurtovic-Kozaric A, Hadziabdic N. 2018. Evaluation of Periodontal Ligament Cell Viability in Different Storage Media Based on Human PDL Cell Culture Experiments - A Systematic Review. Dent Traumatol.

Pavan LM, Rego DF, Elias ST, De Luca Canto G, Guerra EN. 2015. In vitro Anti-Tumor Effects of Statins on Head and Neck Squamous Cell Carcinoma: A Systematic Review. PLoS One. 10.

Pronin S, Koh CH, Hughes M. 2017. Effects of Ultraviolet Radiation on Glioma: Systematic Review. J Cell Biochem. 118:4063–4071.

Rahman NA, Rasil A, Meyding-Lamade U, Craemer EM, Diah S, Tuah AA, Muharram SH. 2016. Immortalized endothelial cell lines for in vitro blood-brain barrier models: A systematic review. Brain Res. 1642:532.

Ramamoorthi M, Bakkar M, Jordan J, Tran SD. 2015. Osteogenic Potential of Dental Mesenchymal Stem Cells in Preclinical Studies: A Systematic Review Using Modified ARRIVE and CONSORT Guidelines. Stem Cells Int. 2015:378368.

Rego DF, Elias ST, Amato AA, Canto GL, Guerra EN. 2017. Anti-tumor effects of metformin on head and neck carcinoma cell lines: A systematic review. Oncol Lett. 13:554.

Richardson DP, Affertsholt T, Asp NG, Bruce A, Grossklaus R, Howlett J, Pannemans D, Ross R, Verhagen H, Viechtbauer V. 2003. PASSCLAIM - Synthesis and review of existing processes. Eur J Nutr.

Rojo R, Prados-Privado M, Reinoso AJ, Prados-Frutos JC. 2018. Evaluation of Fatigue Behavior in Dental Implants from In Vitro Clinical Tests: A Systematic Review. Metals. 8.

Romanelli L, Evandri MG. 2018. Permitted Daily Exposure for Diisopropyl Ether as a Residual Solvent in Pharmaceuticals. Toxicol Res. 34:111–125.

Runkle J, Flocks J, Economos J, Dunlop AL. 2017. A systematic review of Mancozeb as a reproductive and developmental hazard. Environ Int. 99:29–42.

Santos DC, Schneider LR, Silva Barboza A, Diniz Campos Â, Lund RG. 2017. Systematic review and technological overview of the antimicrobial activity of Tagetes minuta and future perspectives. Journal of Ethnopharmacology. 208:8–15.

Sarkis-Onofre R, Skupien JA, Cenci MS, Moraes RR, Pereira-Cenci T. 2014. The role of resin cement on bond strength of glass-fiber posts luted into root canals: a systematic review and meta-analysis of in vitro studies. Oper Dent. 39.

Schneider K, Schwarz M, Burkholder I, Kopp-Schneider A, Edler L, Kinsner-Ovaskainen A, Hartung T, Hoffmann S. 2009. “ToxRTool”, a new tool to assess the reliability of toxicological data. Toxicol Lett. 189:138.

Scholes C, Houghton ER, Lee M, Lustig S. 2015. Meniscal translation during knee flexion: what do we really know? Knee Surg Sports Traumatol Arthrosc. 23:32–40.

Sedrez-Porto JA, Rosa WL, Silva AF, Munchow EA, Pereira-Cenci T. 2016. Endocrown restorations: A systematic review and meta-analysis. J Dent. 52:8–14.

Simko M, Tischler S, Mattsson MO. 2015. Pooling and Analysis of Published in Vitro Data: A Proof of Concept Study for the Grouping of Nanoparticles. Int J Mol Sci. 16:26211–26236.

Simko M, Remondini D, Zeni O, Scarfi MR. 2016. Quality Matters: Systematic Analysis of Endpoints Related to “Cellular Life” in Vitro Data of Radiofrequency Electromagnetic Field Exposure. Int J Environ Res Public Health. 13.

Teixeira MG, Correâ L. 2018. Quality Assessment of Prognostic Studies Using Cancer Stem Cell Markers in Oral Squamous Cell Carcinoma. Applied Immunohistochemistry and Molecular Morphology. 26.

Tsirogiannis P, Reissmann DR, Heydecke G. 2016. Evaluation of the marginal fit of single-unit, complete-coverage ceramic restorations fabricated after digital and conventional impressions: A systematic review and meta-analysis. J Prosthet Dent. 116.

Twetman S, Axelsson S, Dahlen G, Espelid I, Mejare I, Norlund A, Tranaeus S. 2013. Adjunct methods for caries detection: a systematic review of literature. Acta Odontol Scand. 71:388–397.

Vanholder R, Schepers E, Pletinck A, Nagler EV, Glorieux G. 2014. The uremic toxicity of indoxyl sulfate and p-cresyl sulfate: a systematic review. J Am Soc Nephrol. 25:1897.

Verhagen H, Aruoma OI, van Delft JH, Dragsted LO, Ferguson LR, Knasmuller S, Pool-Zobel BL, Poulsen HE, Williamson G, Yannai S. 2003. The 10 basic requirements for a scientific paper reporting antioxidant, antimutagenic or anticarcinogenic potential of test substances in in vitro experiments and animal studies in vivo. Food Chem Toxicol. 41:603–610.

Vesterinen HM, Egan K, Deister A, Schlattmann P, Macleod MR, Dirnagl U. 2011. Systematic survey of the design, statistical analysis, and reporting of studies published in the 2008 volume of the Journal of Cerebral Blood Flow and Metabolism. J Cereb Blood Flow Metab. 31(4):1064–1072.

Xu J, Gong T, Heng BC, Zhang CF. 2017. A systematic review: differentiation of stem cells into functional pericytes. Faseb j. 31:1775–1786.

Yaylali IE, Kececi AD, Ureyen Kaya B. 2015. Ultrasonically Activated Irrigation to Remove Calcium Hydroxide from Apical Third of Human Root Canal System: A Systematic Review of In Vitro Studies. J Endod. 41:1589.

Yosupov N, Haimov H, Juodzbalys G. 2017. Mobilization, Isolation and Characterization of Stem Cells from Peripheral Blood: a Systematic Review. J Oral Maxillofac Res. 8.
